# The null distribution of likelihood-ratio statistics in the conditional-logistic linkage model

**DOI:** 10.3389/fgene.2013.00244

**Published:** 2013-11-19

**Authors:** Yeunjoo E. Song, Robert C. Elston

**Affiliations:** Department of Epidemiology and Biostatistics, Case Western Reserve UniversityCleveland, OH, USA

**Keywords:** linkage analysis, affected sib pairs, identity-by-descent, conditional-logistic model, genetic constraints, null distribution, likelihood-ratio statistics

## Abstract

Olson's conditional-logistic model retains the nice property of the LOD score formulation and has advantages over other methods that make it an appropriate choice for complex trait linkage mapping. However, the asymptotic distribution of the conditional-logistic likelihood-ratio (CL-LR) statistic with genetic constraints on the model parameters is unknown for some analysis models, even in the case of samples comprising only independent sib pairs. We derive approximations to the asymptotic null distributions of the CL-LR statistics and compare them with the empirical null distributions by simulation using independent affected sib pairs. Generally, the empirical null distributions of the CL-LR statistics match well the known or approximated asymptotic distributions for all analysis models considered except for the covariate model with a minimum-adjusted binary covariate. This work will provide useful guidelines for linkage analysis of real data sets for the genetic analysis of complex traits, thereby contributing to the identification of genes for disease traits.

## Introduction

In the study of human data by genetic linkage analysis, the traditional LOD score method, also called a “parametric” or “model-based” method because it requires information about an assumed genetic model, is efficient for single-gene Mendelian traits but is much less well suited for the analysis of traits with complex non-Mendelian modes of inheritance. In the absence of a well-defined disease inheritance model, alternative robust “non-parametric,” “weakly-parametric” or “model-free” linkage methods, which do not require the specification of a disease model, have been used for deciphering the genetic basis of complex traits.

One such approach that has been extremely useful in the analysis of human genetic diseases is the affected sib pair (ASP) study design, as in tests based on the mean proportion of identity-by-descent (IBD) sharing (Blackwelder and Elston, 1985) or tests based on the likelihood-ratio (LR) defined by Risch (1990a,b) that uses the same one-parameter model to analyze ASPs or any other affected unilineal relative pairs by producing a LOD score. Holmans (1993) extended Risch's maximum LOD score method into a two-parameter model for ASPs, but with the genetic constraints required for single locus Mendelian inheritance; here we call this the Risch and Holmans (RH) model. Olson (1999) proposed a general conditional-logistic (CL) model that combines several extensions and modifications (Cordell et al., 1995; Rogus and Krolewski, 1996; Greenwood and Bull, 1997, 1999; Olson, 1997; Lunetta and Rogus, 1998) into a unified framework: the likelihood is conditioned on sampling affected relative pairs (ARPs) and the parameterization is done in terms of the logarithm of allele sharing specific relative risks, instead of allele sharing probabilities as in the RH model. The CL model not only retains the “nice” property of the LOD score formulation of the RH model, i.e., it is additive over independent sets of data, but it also has advantages over the RH model. It is valid for any type of ARPs with the same allele sharing specific parameters. In contrast, the RH model is parameterized in terms of relative-type specific IBD probabilities, so it can accommodate only one ARP type at a time. The other advantage of this CL model is that it can allow for incorporation of covariate effects by re-parameterizing the model in terms of the logarithms of genetic relative risk parameters. A modification of this original two-parameter CL model into a one-parameter model was proposed by Goddard et al. (2001). Linkage analysis using the CL model has been proven to be an effective tool for evaluating genetic linkage (Goddard et al., 2001; Arcos-Burgos et al., 2004; Reck et al., 2005; Doan et al., 2006; Rybicki et al., 2007; Stein et al., 2007; Zandi et al., 2007; Song et al., 2011).

One limitation of the general two-parameter CL model is the unknown asymptotic distribution of certain cases when single-locus genetic constraints are imposed on the model parameters, even in the case of analyzing only independent ASPs. Because of the genetic constraints (Holmans, 1993), the distribution of the CL-LR (i.e., 2ln(10) * LOD score) statistics for linkage are mixtures of χ^2^ distributions that are difficult to specify. The use of simulation methods to obtain *p*-values has been recommended to ensure accuracy of the inference in complex situations (Olson, 1999). Although gene-dropping techniques can be used for this purpose, the ideal method to infer the statistical significance of a test statistic is to compare it with its permutation distribution. When analyzing affected pairs alone, however, permuting the allele sharing of relative pairs does not lead to a useful permutation distribution. As an alternative, Sinha et al. (2006) developed regression prediction models that provide more accurate *p*-values under the CL model framework. However, their results are limited to the cases they evaluated, so it is not a general solution for the unknown distribution of the CL-LR statistic.

Here, we first derive approximations to the asymptotic distributions of the CL-LR statistics when using the constrained two-parameter analysis model for independent ASPs. The derivation is done under the null hypothesis of no linkage and assuming complete marker information, by following Self and Liang (1987), as done for the RH model (Holmans, 1993; Whittemore and Tu, 1998; Feng et al., 2006). Next, we study the empirical null distributions of the CL-LR statistics by simulation, again for independent ASPs, examining several analysis models with different constraints on the model parameters when using the LODPAL program in the S.A.G.E. package (2012). Then, we compare these distributions to the derived asymptotic distributions - either known or approximated in the previous step.

## Materials and methods

### Conditional-logistic model

We first briefly describe the original two-parameter CL model from Olson (1999). The unconditional (prior) probability that a pair of type *r* relatives shares *i* alleles IBD is denoted as *f*_*ri*_, and the estimated probability that the pair shares *i* alleles IBD conditional on the available marker data *I*_*m*_ is denoted as ${\hat{f}}_{ri}$. Then the likelihoods under the null hypothesis (*H*_0_) of no linkage and under the alternative (*H*_1_) can be written as

$$H_{0}:L\left(\lambda_{1}=1,\lambda_{2}=1\right)=P\left(I_{m}|r\right)$$

and

$$H_{1}:L\left(\lambda_{1},\lambda_{2}\right)=P\left(I_{m}|r\right)\frac{\sum_{i=0,1,2}\lambda_{i}{\hat{f}}_{ri}}{\sum_{i=0,1,2}\lambda_{i}f_{ri}},$$

where λ_*i*_ is the relative risk to an individual who shares *i* alleles IBD (*i* = 0, 1, 2) with an affected relative: equating with the notation used in the RH model,λ_0_ = λ_*u*_ (= 1) is the relative risk for unrelated individuals, λ_1_ = λ_*o*_ is the offspring relative risk, and λ_2_ = λ_*m*_ is the MZ-twin relative risk. The CL model is parameterized in terms of the logarithms of relative risk, so λ_*i*_ = e^β*i*^. Under the null hypothesis of no linkage, the parameters (β_1_, β_2_) = (0, 0) correspond to Risch's allele sharing probability parameters (*z*_1_, *z*_2_) = (½, ¼), where *z*_1_ and *z*_2_ are the respective probabilities an ASP shares 1 and 2 alleles IBD at a locus. The LR contribution for an ARP of type *r* is $\text{LR}=\frac{\sum_{i\;=\;012}\lambda_{i}{\hat{f}}_{ri}}{\sum_{i\;=\;012}\lambda_{i}f_{ri}}$, and for a sample of independent ARPs the LOD score is obtained by summing the base-10 logarithms of the pair-specific LRs. For the test of linkage, this LOD score is maximized over a possible range of the parameter space that depends on the constraints imposed, as discussed in the following section. For details of the derivation of the LR and the equivalence of the LR whether the parameterization is in terms of allele sharing probabilities or allele sharing relative risks, we direct the reader to Olson (1999).

When the parameters β_1_ and β_2_ are completely free without any constraints, the parameter space is the whole 2-dimensional plane with two coordinate axes defined by the two parameters. The values of the two parameters under the null hypothesis fall into interior points of this parameter space, and so the CL-LR statistic under the null hypothesis of no linkage is distributed as χ^2^_2_ asymptotically. We refer to this model as the *unconstrained two-parameter model*.

When the (pure single-locus etiology) genetic constraints (Holmans, 1993) are imposed, the parameter β_1_ and β_2_ are constrained to be β_1_ ≥ 0 and β_2_ ≥ log_*e*_ (2*e*^β^_1_ − 1), or equivalently, λ_1_ ≥ 1 and λ_2_ ≥ 2λ_1_ − 1, to reflect the possible allele sharing probabilities for ASPs. In this case, the values of the parameters under the null hypothesis are on the edge of the parameter space, so that the LR statistic is asymptotically distributed as the mixture $\left(\frac{1}{2}-c\right)\chi_{0}^{2}+\frac{1}{2}\chi_{1}^{2}+c\chi_{2}^{2}$ with the mixing proportion *c* representing the probability that the allele sharing estimates fall inside a triangle that is part of the two-dimensional plane. We refer to this model as the *constrained two-parameter model*.

### Mixing proportion c

The mixing proportion *c* is a function of the expected information matrix. For the RH model with allele sharing parameters, it has been derived to be *c* ≈ 0.098 when there is complete marker information (Holmans, 1993; Whittemore and Tu, 1998; Feng et al., 2006), regardless of the choice of any two free parameters, i.e., (*z*_0_, *z*_1_), (*z*_0_, *z*_2_), or (*z*_1_, *z*_2_). However, for the CL model with the parameters in terms of the logarithms of relative risk, this value is unknown. We apply the method of Self and Liang (1987), as for the RH model, to derive the mixing proportion *c* for the LR statistic in the CL genetic constrained, two-parameter model.

**Figure 1:**
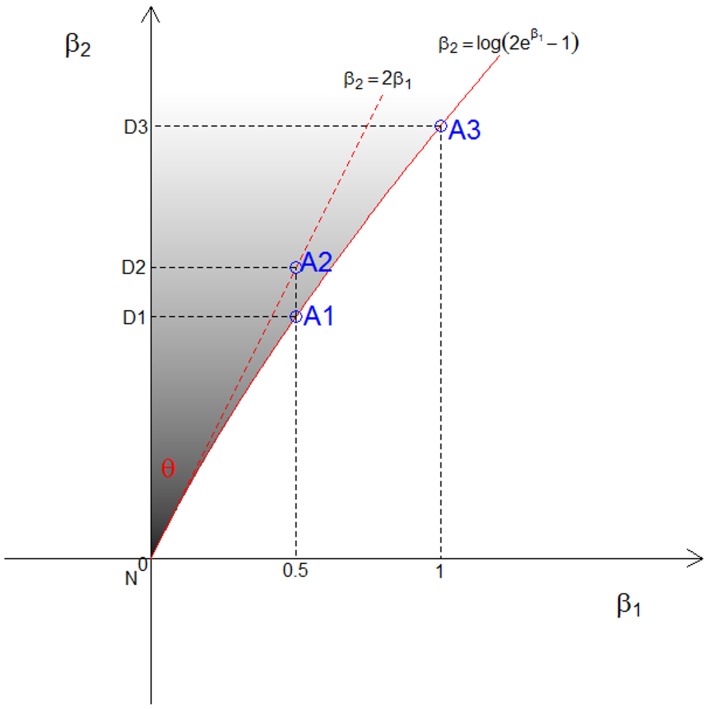
**The three points (A1, A2, and A3) used to approximate the relation between β_1_ and β_2_ and the upper bound of β_1_ under genetic constraints in the CL model**. The corresponding dominant points are denoted (D1, D2, and D3), and the shaded area is the possible triangle area in the CL model.

As shown in Figure 1, let (β_1_,β_2_) represent a point in the 2-dimensional plane with two coordinate axes that are defined by the parameters β_1_ and β_2_, constrained to be β_1_ ≥ 0, β_2_ ≥ log_*e*_ (2*e*^β^_1_ − 1) (gray area). We first define the three vertices of possible triangles in the (β_1_,β_2_) plane. Let *N* = (0, 0) be the null point, *A* denote an additive inheritance point, and *D* a dominant inheritance point. The point *A* will be on the line β_2_ = log_*e*_ (2*e*^β^_1_ − 1). We define *D* = (0, β_2_) as a point on the β_2_ axis where the value of β_2_ is the same as the point *A*, as in Figure 1. Let *I* be the Fisher information matrix of the likelihood function *L*(*data*|${\hat{\beta }}_{1}$, ${\hat{\beta }}_{2}$) evaluated at the null values. Assuming complete information, the variance-covariance matrix of the parameters is the inverse of *I*, i.e., $I^{-1}=\left(\begin{array}{cc} 6 & 4\\ 4 & 8 \end{array}\right)$. Let PΛP^*T*^ be the spectral decomposition of *I*^−1^, and *Y*_*N*_, *Y*_*A*_, and *Y*_*D*_ be the orthogonally transformed vertices of *N*, *A* and *D* such that $Y=\Lambda^{1/2}{\text{P}}^{T}\left(\hat{\beta }-N\right)$. Let *y*_*N*_, *y*_*A*_, and *y*_*D*_ be the rotated vertices of *Y*_*N*_, *Y*_*A*_ and *Y*_*D*_ such that *Y*_*A*_ lies on the β_1_ axis and the ray defined by two points *Y*_*N*_ and *Y*_*D*_ becomes the hypotenuse in the upper right quadrant of the plane. Now, the three rotated vertices *y*_*N*_, *y*_*A*_, and *y*_*D*_ define the triangle area in the orthogonal space, and the angle θ formed by the two rays $\vec{y_{N}y_{A}}$ and $\vec{y_{N}y_{D}}$ represents the mixing proportion *c*. Letting the end point of the hypotenuse be (*x*, *y*), $\theta =arctan\left(\frac{y}{x}\right)$ and $c=\frac{\theta }{\text{2}\pi }$.

If a model with no dominance genetic variance is to fit, then β_2_ = log_*e*_ (2*e*^β^_1_ − 1), as shown by a solid red line in Figure 1. Owing to the fact that this line is not straight, the angle θ differs according to the choice of the point *A* on the line. The point *A* depends on both the assumption we make about the relation between β_1_ and β_2_, and the upper value of β_1_ that is chosen. We consider 3 different points for *A*, denoted A1, A2, and A3, as shown in Figure 1. First, under the A1 assumption, we take the exact relation between β_1_ and β_2_, i.e., β_2_ = log_*e*_(2*e*^β^_1_ − 1), and approximate the angle θ under the assumption that β_1_ represents the allele sharing probability *z*_1_, which has maximum value ½. Second, with the A2 assumption, we approximate a straight line about the null value using a Taylor series expansion, i.e., β_2_ = 2β_1_ (dotted red line in Figure 1). In this case, the upper bound of β_1_ is irrelevant. This is equivalent to using the triangle obtained from the constraints on λ, i.e., λ_2_ = 2λ_1_ − 1. Third, with the A3 assumption, we take the exact relation between β_1_ and β_2_ and approximate the angle θ under the assumption that β_1_ can go up to 1. This is equivalent to assuming the maximum offspring relative risk λ_1_ = λ_0_ ≈ 2.718. We derive the resulting mixing proportions for these 3 cases and expand them for more values in the results section.

### One-parameter model

Goddard et al. (2001) proposed to modify the two-parameter model into a one-parameter model on the basis of the minmax model developed by Whittemore and Tu (1998). In this one-parameter model, the constraint λ_2_ = (π + 1)λ_1_ − π was imposed, where π is a parameter associated with the mode of inheritance and is fixed to be 2.634, i.e., β_2_ = log_*e*_ (3.634*e*^β^_1_ − 2.634) (Olson, 2002). This constraint assumes a genetic model approximately halfway between a recessive and a dominant mode of inheritance, which has been shown to be usually more powerful for most genetic models.

For this one-parameter model, the CL-LR statistic is known to be asymptotically distributed as a χ^2^_1_ when β_1_ is free without any constraints, because its null value is an interior point of the parameter line. Even though Whittemore and Tu's minmax constraint is already imposed to make it a one-parameter model, we refer to this model as the *unconstrained one-parameter model* because β_1_ is completely free without any genetic constraints. When the parameter space for β_1_ is constrained by β_1_ ≥ 0 (equivalently λ_1_ ≥ 1) to reflect non-negative allele sharing probabilities, the CL-LR statistic is asymptotically distributed as a 50:50 mixture of a point mass at 0 and χ^2^_1_. We refer to this as the *constrained one-parameter model*.

### Covariates

If there are *K* covariates in the model, assuming a log-linear (i.e., multiplicative) effect of the covariate on genetic relative risk, which is a common, natural, and flexible way to model relative risk in general epidemiology (Olson, 1999), the relative risk is λ_*i*_ = exp(β_*i*_ + ∑^*K*^_*j* = 1_δ_*ij*_*x*_*j*_), where the δ_*ij*_ are the two parameters associated with the covariate *x*_*j*_, with β_0_ = δ_0*j*_ = 0. Therefore, each covariate added requires two additional parameters for the two-parameter model but only one additional parameter for the one-parameter model.

When there are no constraints imposed on the covariate parameters, with the addition of *K* covariates the CL-LR statistic is asymptotically distributed as χ^2^_2(*k* + 1)_ in the unconstrained two-parameter model. For the triangle-constrained two-parameter model, with the addition of *K* covariates the distribution of the CL-LR statistic is a mixture of a point mass at 0 and several χ^2^ s with up to 2(*K* + 1) df, asymptotically. However, no covariates are allowed in the two-parameter model in the LODPAL program in the S.A.G.E. package (2012), owing to the practical difficulty of maximizing the likelihood of models with two additional parameters for each covariate. Therefore, in this study we did not consider the two-parameter models with covariates.

For the one-parameter model, addition of covariates requires one additional parameter for each covariate. With the addition of K covariates, without any additional constraints imposed on covariate parameters the CL-LR statistic is asymptotically distributed as χ^2^_*k* + 1_ in the unconstrained one-parameter model. Addition of *K* covariates in the constrained one-parameter model, again without any additional constraints imposed on the covariate parameters, gives a CL-LR statistic with a distribution that is asymptotically a 50:50 mixture of a χ^2^ with *K* df and a χ^2^ with *K* + 1 df, (Goddard et al., 2001). In this study, we only included the constrained one-parameter model with covariate(s), and this is referred to as the *covariate model*.

Depending on additional constraints on the covariates, we define two covariate models. By including a “mean-centered” covariate (*x* − *x*), no constraints on the δ_1*j*_ are required (Olson, 1999), so the CL-LR statistic is asymptotically distributed as a 50:50 mixture of two χ^2^ s depending on the number of such covariates, as stated previously. This is reasonable for many covariates, in particular continuous covariates such as age. We refer to this as the *unconstrained covariate model*.

However, for some covariates, such as indicator variables that represent different populations or a binary factor, the offset from the minimum value of the covariate, i.e., “minimum-adjusted,” [*x*_*a*_ = *x* − min(*x*)] is included in the model, so that the smallest value of the covariate equals zero. For such covariates, the constraint $\underset{x_{aj}>0}{\min}\sum_{j}x_{aj}\delta_{1j}\geq -\beta_{1}$ is applied; it is not then feasible to derive the asymptotic distribution of the CL-LR statistic under the null hypothesis theoretically, since it depends on the distribution of the covariate values in the given data. We refer to this as the *constrained covariate model*.

### Simulations

To examine the precision of the expected asymptotic distributions in the previous section, we used simulation to determine the empirical null distributions of the CL-LR statistics. We considered 6 different analysis models described in the previous section. We considered the covariate model with just one covariate. For the unconstrained covariate model, we included one with a mean-centered continuous covariate. For the constrained covariate, we included one with a minimum-adjusted binary covariate.

We first simulated 100,000 replicates of 500 nuclear families having two parents and two affected siblings, i.e., 500 independent ASPs. For each case, one fully informative unlinked marker was simulated by assigning a unique allele to each founder, and then the alleles were randomly segregated to all offspring. For covariate models, under the null hypothesis of no linkage and no covariate effect, the covariate was simulated such that it was correlated with affection status but not with genotype. A random continuous value from a normal distribution with mean 0 and variance 1 was first assigned to each individual, regardless of affection status. Then a continuous covariate was simulated by adding a pre-fixed covariate effect to this value. A binary covariate was generated by dichotomizing this continuous covariate such that its population prevalence was 0.2. Given the covariate values for each member of the pair, the pair-level covariate for a pair was created by summing the two individual-level covariates. The continuous pair-wise covariate values for the unconstrained covariate model are mean-centered, and the binary pair-wise values for the constrained covariate model are minimum-constrained when they are included in the analysis.

To check the performance of the expected asymptotic null distribution for each analysis model under different sample sizes, we also simulated 100,000 replicates of 30, 50, and 100 families, as above. Additionally, the precision of the approximated asymptotic null distributions of the CL-LR statistics for the constrained two-parameter model was compared with the empirical null distributions under different marker information levels. We simulated 100,000 replicates of 100 independent ASPs for markers with 2, 4, 8, and 20 equally frequent alleles. These numbers correspond to PIC values of 0.38, 0.70, 0.86, and 0.95, respectively. We checked two cases, when both parents are typed and when neither is typed.

The empirical *p*-value corresponding to the LOD score was determined by assigning *p* = (*r* + 1)/(100,000 + 1) to the *r*th of the ranked LOD scores from 100,000 replicates. The asymptotic *p-value* corresponding to the same LOD score was calculated using the known or approximated asymptotic distribution, as described above.

## Results

### Asymptotic null distributions under triangle constraints

**Figure 2:**
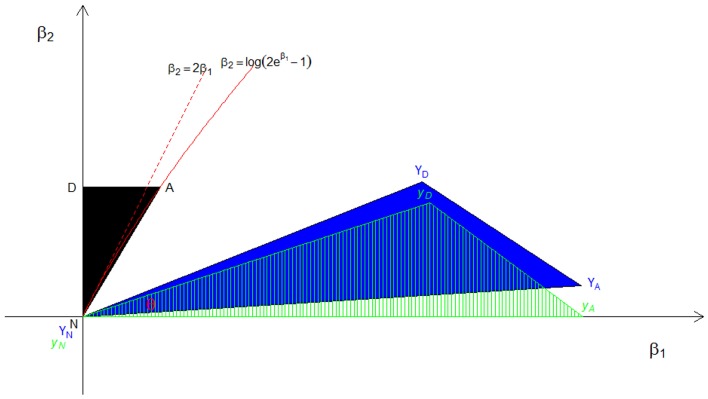
**The distribution of constrained CL-LR statistics under the A1 approximation**. The black area (*N*, *A*, and *D*) is the original possible triangle space for ASPs, the blue area (*Y*_*N*_, *Y*_*A*_, and *Y*_*D*_) is the orthogonally transformed triangle, and the green dashed triangle (*y*_*N*_, *y*_*A*_, and *y*_*D*_) is the space after rotation. The angle θ formed by the two rays *y*_*N*_ *y*_*A*_ and *y*_*N*_ *y*_*D*_ represents the mixing probability *c*.

The resulting triangles under assumption A1 are graphically illustrated in Figure 2, showing the steps to derive the mixing proportion for a given value of *A*. In this figure, the possible triangle space for ASPs on the original (β_1_, β_2_) plane is in black, formed by the three vertices (N, A, D) = {[0, 0], [½, log_*e*_(2*e*^1/2^ − 1)], [0, log_*e*_(2*e*^1/2^ - 1)]}. Then, we have

$$\begin{array}{l} Y_{N}={\left(\begin{array}{cc} 11.12 & 0\\ 0 & 2.88 \end{array}\right)}^{1/2}{\left(\begin{array}{cc} 0.615 & -0.788\\ 0.788 & 0.615 \end{array}\right)}^{T}\left(\begin{array}{c} 0\\ 0 \end{array}\right)=\left(\begin{array}{c} 0\\ 0 \end{array}\right),\\ Y_{A}={\left(\begin{array}{cc} 11.12 & 0\\ 0 & 2.88 \end{array}\right)}^{1/2}{\left(\begin{array}{cc} 0.615 & -0.788\\ 0.788 & 0.615 \end{array}\right)}^{T}\left(\begin{array}{c} 0.5\\ \log_{e}\left(2e^{0.5}-1\right) \end{array}\right)\\ \;=\left(\begin{array}{l} 3.213\\ 0.199 \end{array}\right), \end{array}$$

$$\begin{array}{l} Y_{D}={\left(\begin{array}{cc} 11.12 & 0\\ 0 & 2.88 \end{array}\right)}^{1/2}{\left(\begin{array}{cc} 0.615 & -0.788\\ 0.788 & 0.615 \end{array}\right)}^{T}\left(\begin{array}{c} 0\\ \log_{e}\left(2e^{0.5}-1\right) \end{array}\right)\\ \;=\left(\begin{array}{l} 2.187\\ 0.868 \end{array}\right); \end{array}$$

and then $y_{N}=\left(\begin{array}{c} 0\\ 0 \end{array}\right),y_{A}=\left(\begin{array}{c} 3.219\\ 0 \end{array}\right)$, and $y_{D}=\left(\begin{array}{c} 2.236\\ 0.731 \end{array}\right)$.

The corresponding orthogonally transformed triangle (*Y*_*N*_, *Y*_*A*_, *Y*_*D*_) is in blue, and the green dashed triangle (*y*_*N*_,*y*_*A*_,*y*_*D*_) is the same orthogonally transformed triangle after rotation such that *Y*_*A*_ lies on the β_1_ axis and the ray defined by *Y*_*N*_ and *Y*_*D*_ becomes the hypotenuse in the upper right quadrant of the plane. Then the angle θ formed by the two rays $\vec{y_{N}y_{A}}$ and $\vec{y_{N}y_{D}}$ in the green triangle is $arctan\left(\frac{0.731}{2.236}\right)\approx 0.316$, and the corresponding mixing proportion *c*_1_ is $\frac{\theta }{\text{2}\pi }\approx 0.050$. By following the same steps, we find the mixing proportions to be *c*_2_ ≈ 0.044 and *c*_3_ ≈ 0.054, respectively, under the A2 and A3 assumptions.

**Figure 3:**
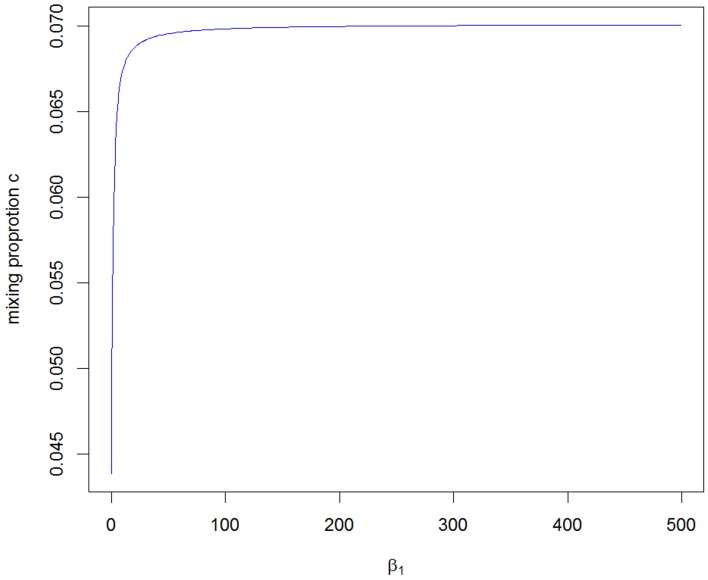
**The range of the mixing proportion values according to the different beta1 values for the distribution of the CL-LR statistics from the constrained two-parameter model**.

The value of *c*_2_ obtained from the A2 assumption provides the minimum bound for *c* and, from the A1 and A3 assumptions, we can see that the mixing proportion value *c* becomes larger as we take a larger upper value for β_1_. Figure 3 shows how the value of *c* depends on the value of the parameter β_1_. It can be seen that the maximum value converges to around 0.070, which is smaller than the value for the RH model. The critical LOD score values corresponding to the test sizes 0.05, 0.01, 0.001, 0.0001 [the classical “LOD score 3” criterion given by Morton (1955)], 0.000049 [significant evidence for linkage given by Lander and Kruglyak (1995)] and 0.00001 are given in Table 1 for the different mixing proportion values. Given the same size of test, the critical LOD scores for the CL model are smaller than those for the RH model. Therefore, the null hypothesis is more likely to be rejected using the CL-LR test, and the CL-LR statistic is more powerful.

**Table 1:** **Critical LOD scores obtained from the constrained two-parameter models for different mixing proportion values; *CL* − *c*_*min*_ and *CL* − *c*_*max*_ are the minimum and maximum *c* values for the CL model, *A1-c* is the value from the A1 approximation, and *RH-c* is the mixing proportion for the RH model**.

**Mixing proportion**	**Size of test**					
	**0.05**	**0.01**	**0.001**	**0.0001**	**0.000049**	**0.00001**
*CL-c*_*min*_	0.662	1.276	2.202	3.154	3.452	4.118
*A1-c*	0.672	1.289	2.219	3.172	3.470	4.138
*CL-c*_*max*_	0.702	1.328	2.265	3.225	3.524	4.195
*RH-c*	0.742	1.377	2.324	3.290	3.591	4.265

### Empirical null distributions

#### Two-parameter model

**Figure 4:**
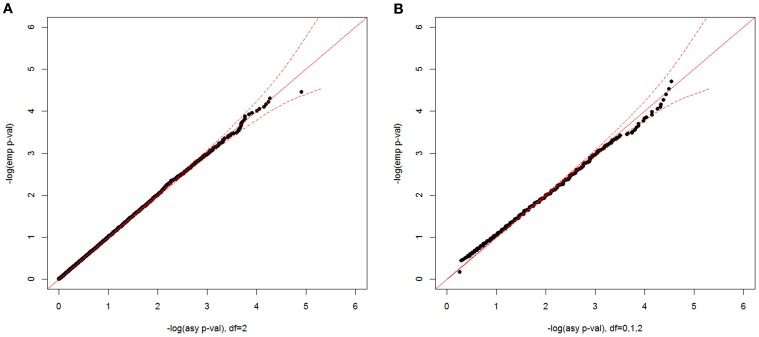
**Null distributions of the CL-LR statistics for the two-parameter models, using 500 independent ASPs and a fully informative marker**. The empirical *p*-values for the observed LR statistics (y-axis) are plotted against the asymptotic *p*-values from known chi-square distribution (x-axis) for the unconstrained model **(A)** and for the constrained model **(B)** Note that the asymptotic distribution for the constrained model is under the A1 assumption, and a 95% confidence interval is shown by the dotted red line.

In Figure 4, we show plots of –log_10_(empirical *p*-value) against –log_10_(asymptotic *p*-value) corresponding to the observed CL-LR statistics with a sample size 500 for two two-parameter models. For the unconstrained model, the empirical *p*-values well matched the asymptotic *p*-values from the expected chi-square distribution with 2 df. For the constrained model, the mixture distribution from the A1 assumption was also close to the empirical distribution. Since the mixing proportions from the three approximations are so close to each other, the empirical distributions matched the asymptotic distributions well for all three different mixing proportions (results not shown).

**Figure 5:**
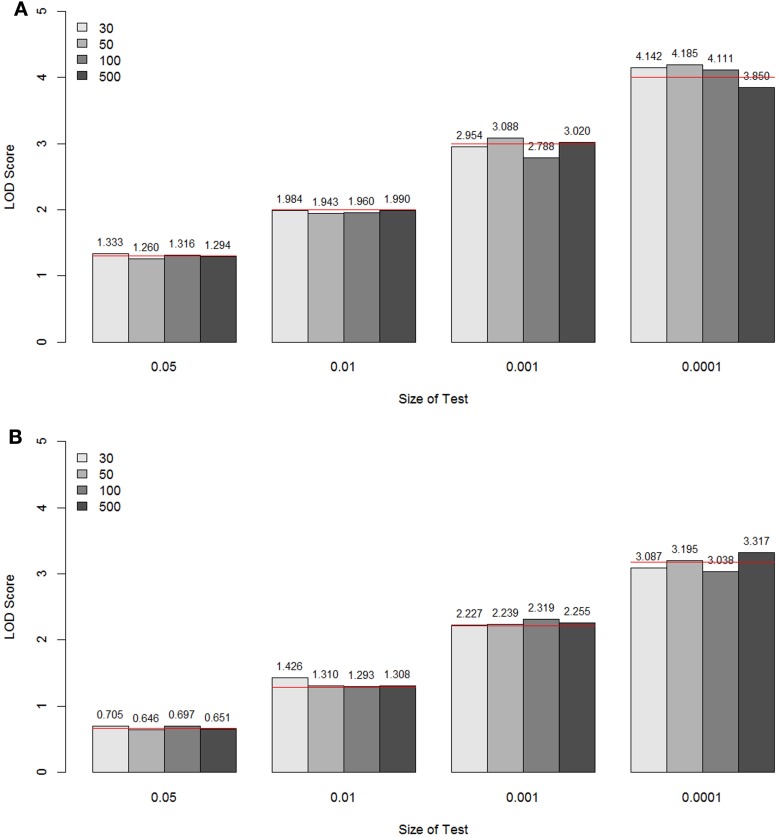
**The LOD score values corresponding to the empirical *p*-values 0.05, 0.01, 0.001, and 0.0001 for the unconstrained two-parameter model (A) and the constrained two-parameter model (B), by sample size and size of the test**. These values are the critical values for the type I error rates equal to the given empirical p-values. The theoretical values are shown as a red line for each *p*-value.

For each sample size simulated, the specific LOD score values corresponding to the empirical *p*-values 0.05, 0.01, 0.001, and 0.0001 for these two models are given in Figure 5, compared with the theoretical values (shown as a red line for each *p*-value). These values are the critical values for the type I error rates equal to the given empirical p-values. Overall, for all sample sizes, the critical LOD scores from the empirical distributions were similar and very close to the values from the asymptotic distributions, well up to about –log_10_(*p*-value) = 3. When the type I error rate is 0.0001, the critical LOD scores varied depending on the sample size.

**Figure 6:**
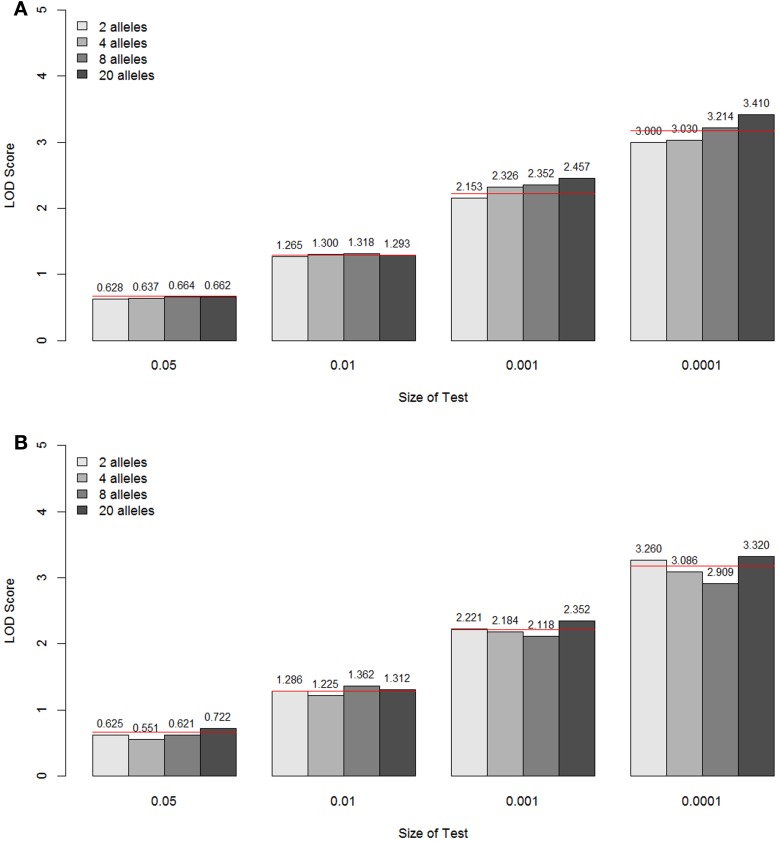
**LOD score values corresponding to the empirical *p*-values 0.05, 0.01, 0.001, and 0.0001 under different marker information levels for the constrained two-parameter model, when the parents are typed (A) and not typed (B)**. These values are the critical values for the type I error rates equal to the given empirical p-values. The theoretical values are shown as a red line for each *p*-value.

The empirical null distributions under different marker information levels for the constrained two-parameter model are shown in Figure 6 (A for parents typed, B for parents not typed). For the two types of parental information, the specific LOD score values corresponding to the empirical *p*-values 0.05, 0.01, 0.001, and 0.0001 are again compared with the theoretical values from the A1 assumption (shown as a red line for each *p*-value). Again, it can be seen that the approximated asymptotic null distribution well matched the empirical distribution for the different levels of marker information, both in terms of the number of alleles and the amount of parental information.

#### One-parameter model

Here again, we found that the distribution of LOD scores follows the theoretical distribution well (results not shown). For both one-parameter models, the empirical *p*-values well matched the asymptotic *p*-values from the expected chi-square distributions. For the unconstrained case, the CL-LR statistic was distributed as a χ^2^_1_, as expected. The empirical distribution of the CL-LR statistics for the constrained model followed closely a 50:50 mixture of a point mass at 0 and a χ^2^_1_, which again agrees with the asymptotic distribution. For all sample sizes, the critical LOD scores from the empirical distributions were again similar and very close to the values from the asymptotic distributions well, up to about -log_10_ (*p*-value) = 3, and they varied depending on the sample size when the type I error rate is 0.0001, as for the two-parameter model.

#### Covariate model

**Figure 7:**
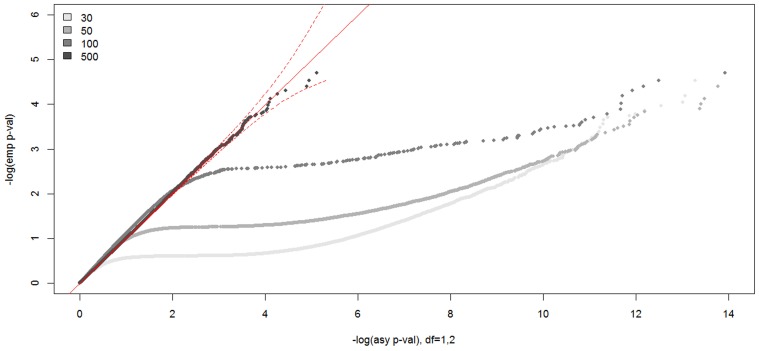
**Null distributions of the CL-LR statistics for the unconstrained covariate models, using 30, 50, 100, and 500 independent ASPs and a fully informative marker**. The empirical *p*-values for the observed LR statistics (*y*-axis) are plotted against the asymptotic *p*-values from the known chi-square distribution (*x*-axis) for the unconstrained covariate model. The dotted red line is the 95% confidence interval.

In Figure 7, we show the distributions of empirical *p*-values under the null hypothesis of no linkage for the unconstrained covariate model. The empirical *p*-values for the covariate model with one unconstrained continuous covariate matched well the asymptotic *p*-values from a 50:50 mixture of a χ^2^_1_ and a χ^2^_2_ distribution when the sample size was 500, as expected. However, unlike other analysis models, the distribution of LOD scores did not follow the theoretical distribution for the smaller sample sizes. We found the empirical null distribution departed more from the asymptotic null distribution the smaller the sample size, as expected. For example, the critical LOD scores were over 10.0 for sample sizes 30, 50, and 100, compared to 3.77 from the asymptotic distribution for the test size 0.0001.

**Figure 8:**
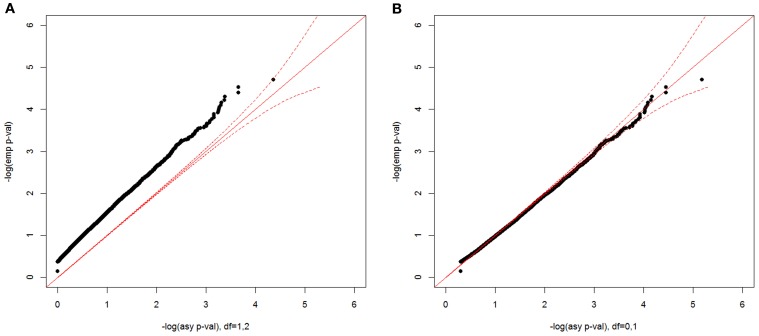
**Null distributions of the CL-LR statistics for the constrained covariate model, using 500 independent ASPs and a fully informative marker**. The empirical *p*-values for the observed LR statistics (y-axis) are plotted against the asymptotic *p*-values from a 50:50 mixture of a χ^2^_1_ and a χ^2^_2_ distribution **(A)**, and from a 50:50 mixture of a point mass at 0 and a χ^2^_1_ **(B)** The dotted red line is the 95% confidence interval.

For the constrained covariate model with a minimum-adjusted binary covariate, we show the empirical null distribution compared with two asymptotic distributions in Figure 8, one with a 50:50 mixture of a χ^2^_1_ and a χ^2^_2_ distribution (A) and the other with a 50:50 mixture of a point mass at 0 and χ^2^_1_ distribution (B). The asymptotic *p*-values from a 50:50 mixture of a χ^2^_1_ and a χ^2^_2_ distribution were too conservative, while the asymptotic *p*-values from a point mass at 0 and χ^2^_1_ distribution well matched the empirical *p*-values. In the simulated data for this model, the possible pair-wise covariate values are 0, 1, or 2, since we included the sum of two individual binary covariate values. Since β_1_ ≥ 0 and $\underset{x_{aj}>0}{\min}\sum_{k}x_{aj}\delta_{k}\geq -\beta_{1}$, δ_1_ ≥ 0 when β_1_ = 0. When β_1_ > 0, the minimum value of δ_1_ is $\frac{-\beta_{1}}{2}$. Therefore, the two-parameter space is constrained to be 1/3 of the whole plane, instead of 1/2 of the plane, which causes the asymptotic *p*-values from a 50:50 mixture of a χ^2^_1_ and a χ^2^_2_ distribution to be too conservative. In practice, the distribution will depend on the distribution of the covariate values in the data.

**Figure 9:**
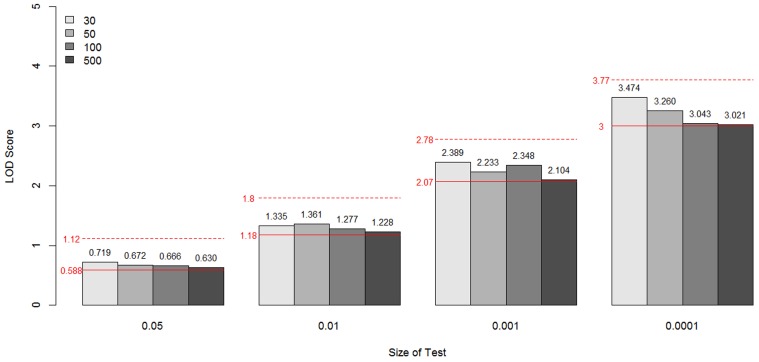
**LOD scores corresponding to the empirical *p*-values 0.05, 0.01, 0.001, and 0.0001 for the constrained covariate model by sample size and size of test**. These values are the critical values for the type I error rates equal to the given empirical p-values. The theoretical values are shown as a red line for each *p*-value. The dotted lines are from a 50:50 mixture of a χ^2^_1_ and a χ^2^_2_ distribution and the solid lines are from a 50:50 mixture of a point mass at 0 and a χ^2^_1_.

In Figure 9, the specific LOD score values corresponding to the empirical *p*-values 0.05, 0.01, 0.001, and 0.0001 are given for each sample size simulated. These values are again the critical values for the type I error rates equal to the given empirical p-values, compared with theoretical values (shown as a red line for each *p-value)*. The dotted lines are from a 50:50 mixture of a χ^2^_1_ and a χ^2^_2_ distribution, and the solid lines are from a 50:50 mixture of a point mass at 0 and a χ^2^_1_.

## Discussion

In the RH model, the mixing probability *c* (which represents the probability that the allele sharing estimates fall inside the possible triangle) is the same for any two allele-sharing parameters. However, this is not so in the CL model owing to the non-straight line relation between the two parameters β_1_ and β_2_, the logarithms of relative risks. In this paper, we developed three approximations to the asymptotic distributions of the CL-LR statistics for the constrained two-parameter model, under the null hypothesis of no linkage, for independent ASPs. We derived the mixing probability *c* assuming complete information, as was done for the RH model with Risch's allele sharing parameters, following the method given by Self and Liang (1987). From these three approximations, we also investigated the relation between the parameter values for β_1_ and *c*. We found the range of the *c* values to be (0.0439–0.070), which is lower than the value obtained for the RH model. This results in critical LOD score values lower by 5–11% (0.702–0.662 vs. 0.742) for a test size 0.05, and by 3–5% (2.265–2.202 vs. 2.324) for a test size 0.001, compared to the RH model. Therefore, the test using the CL-LR statistic will be more powerful, though perhaps not significantly so. In practice, the estimate of β_1_ can be used to decide on an appropriate value for *c* to obtain a reasonably accurate test of linkage for a particular set of data.

By simulation, the performance of the approximate asymptotic distribution was checked for various sample sizes both when there is perfect information and under different marker information levels. This was done for two different parental information cases (typed and not typed) for a fixed sample size of 100 independent ASPs. Generally, for all sample sizes and the different levels of information content investigated, we found the empirical null distribution of the CL-LR statistic from the constrained two-parameter model matches well the approximated asymptotic distribution. This result shows the applicability of the approximated asymptotic distribution to real data analysis for any marker.

For the unconstrained two-parameter model, the unconstrained one-parameter model, and the constrained one-parameter model, we also found that the known asymptotic distributions matched the empirical distributions well. Therefore, for these models, the test of linkage using the CL-LR statistic can be performed using the known asymptotic null distribution to find the *p*-value. The unconstrained models may not be biologically plausible, but could be useful for the purpose of comparison, or when the data include ASPs with a different direction of genetic effect caused by other factors, as investigated by Dizier et al. (2000).

Unlike for the other models, a large sample size was needed for the asymptotic distribution to hold well for the unconstrained covariate model, i.e., the constrained one-parameter model with an unconstrained covariate. Sinha et al. (2006) also reported this vast discrepancy between the asymptotic *p*-values and the empirical *p*-values for this model. Their result was based on average sample sizes of 20, 40, 80, 120, and 320 affected pairs. To determine the sample size necessary for the asymptotic *p*-values to be applicable, we additionally simulated 200 and 300 ASPs. This showed that with 200 ASPs the empirical distribution matched well the asymptotic distribution (results not shown). Therefore, in practice, for this model we recommend the use of simulation methods or the Sinha et al. method when the sample size is less than 200, to ensure accurate *p*-values.

Though the results are not shown, from additional simulations with two and three covariates and 500 ASPs, except in the tail, the distributions of CL-LR statistics for the unconstrained covariate model with two covariates also closely matched a 50:50 mixture of a χ^2^_2_ and a χ^2^_3_, and that for three covariates a 50:50 mixture of a χ^2^_3_ and a χ^2^_4_, as expected from the asymptotic distributions. These results confirm that the empirical distribution of the CL-LR statistic for comparing nested unconstrained covariate models that differ by *J* covariates has a χ^2^ distribution with *J* df, as expected from the asymptotic distribution. Therefore, in large samples it is valid to test the significance of the contribution of a covariate using the asymptotic distribution.

**Figure 10:**
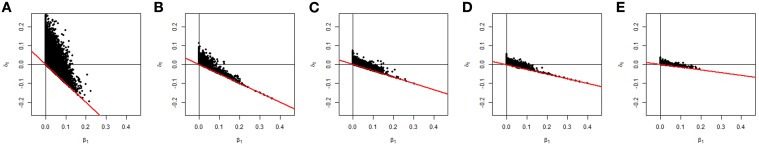
**Distributions of the estimates of β_1_ and δ_1_ under the constrained covariate model for different covariate distributions**. The covariate values were (0 or 1) **(A)**, (0, 1 or 2) **(B)**, (0, 1, 2, or 3) **(C)**, (0, 1, 2, 3, or 4) **(D)** and a random number in the range (0, 8) **(E)**

It was interesting to find in our simulated data that the empirical null distribution for the constrained covariate model, i.e., constrained one-parameter model with a constrained covariate, was closer to a 50:50 mixture of a point mass at 0 and χ^2^_1_ distribution than to a 50:50 mixture of a χ^2^_1_ and a χ^2^_2_ distribution. This is due to the functional dependency of δ_1_ on the maximum covariate value in the data when β_1_ > 0. This dependency effectively reduces the degrees of freedom and hence changes the distribution. To show how the range of the covariate values in the data changes the null values of the parameters, and therefore the distribution of the CL-LR statistics, we additionally simulated datasets with pair-wise covariate values (0 or 1), (0, 1, 2, or 3), (0, 1, 2, 3, or 4), and a random number in the range (0, 8). In Figure 10, we show a plot of the estimates of the parameters β_1_ and δ_1_, including the result from the (0, 1, or 2) case in the previous simulation. We can see that the space for two parameters becomes smaller as the maximum value of the minimum-adjusted covariate increases. For the (0 or 1) case, it seems the CL-LR statistics will be closely distributed as the mixture *c*_0_ χ^2^_0_ + *c*_1_ χ^2^_1_ + *c*_2_ χ^2^_2_. In other cases, a 50:50 mixture of a point mass at 0 and χ^2^_1_ distribution closely matched the empirical distribution. Therefore, in practice, the distribution will depend on the distribution of the covariate values in the dataset, so careful examination of the distributions of the covariates in the dataset is needed before including them in any analysis.

We did not include any power analysis in this study because our purpose was to find an approximation to the theoretically unknown null distributions and to compare them with the empirical null distribution, to provide guidelines for testing linkage when using the CL-LR statistics in various analysis models. To our knowledge, there has not been any study of the null distribution of LOD scores for the CL model, neither theoretical nor empirical. The results from this study should provide useful guidelines for the linkage analysis of real datasets since our results are based on both a perfect scenario as well as on non-perfect cases. Our results for various sample sizes will also provide guidelines for cases with missing data, since these will in general correspond to a reduced sample size. We assumed no errors in the relationship between pairs. When the information content in the marker and/or pedigree structure in real data are reduced due to errors in the data, we would generally expect the power to be lower for given type I error; but the test of linkage based on our results will still be valid, as long as the analysis is done on independent pairs.

### Conflict of interest statement

The authors declare that the research was conducted in the absence of any commercial or financial relationships that could be construed as a potential conflict of interest.
